# A New Master Donor Virus for the Development of Live-Attenuated Influenza B Virus Vaccines

**DOI:** 10.3390/v13071278

**Published:** 2021-06-30

**Authors:** Chantelle L. White, Kevin Chiem, Daniel R. Perez, Jefferson Santos, Stivalis Cardenas Garcia, Aitor Nogales, Luis Martínez-Sobrido

**Affiliations:** 1 Department of Microbiology and Immunology, University of Rochester, Rochester, NY 14625, USA; Chantelle_White@URMC.Rochester.edu (C.L.W.); kchiem@txbiomed.org (K.C.); nogales.aitor@inia.es (A.N.); 2 Texas Biomedical Research Institute, San Antonio, TX 78245, USA; 3 Poultry Diagnostic and Research Center, Department of Population Health, University of Georgia, Athens, GA 30602, USA; dperez1@uga.edu (D.R.P.); jefferson.jss@gmail.com (J.S.); stivalis@uga.edu (S.C.G.); 4 Centro de Investigación en Sanidad Animal (INIA-CISA), Instituto Nacional de Investigación y Tecnología Agraria y Alimentaria, 28130 Madrid, Spain

**Keywords:** influenza, influenza B virus, vaccines, live-attenuated influenza vaccines, safety, immunogenicity, protection efficacy, master donor virus, temperature sensitive, cold-adapted, attenuated

## Abstract

Influenza B viruses (IBV) circulate annually, with young children, the elderly and immunocompromised individuals being at high risk. Yearly vaccinations are recommended to protect against seasonally influenza viruses, including IBV. Live attenuated influenza vaccines (LAIV) provide the unique opportunity for direct exposure to the antigenically variable surface glycoproteins as well as the more conserved internal components. Ideally, LAIV Master Donor Viruses (MDV) should accurately reflect seasonal influenza strains. Unfortunately, the continuous evolution of IBV have led to significant changes in conserved epitopes compared to the IBV MDV based on B/Ann Arbor/1/1966 strain. Here, we propose a recent influenza B/Brisbane/60/2008 as an efficacious MDV alternative, as its internal viral proteins more accurately reflect those of circulating IBV strains. We introduced the mutations responsible for the temperature sensitive (ts), cold adapted (ca) and attenuated (att) phenotype of B/Ann Arbor/1/1966 MDV LAIV into B/Brisbane/60/2008 to generate a new MDV LAIV. In vitro and in vivo analysis demonstrated that the mutations responsible of the ts, ca, and att phenotype of B/Ann Arbor/1/1966 MDV LAIV were able to infer the same phenotype to B/Brisbane/60/2008, demonstrating its potential as a new MDV for the development of LAIV to protect against contemporary IBV strains.

## 1. Introduction

Over the last century, influenza viruses have been responsible of both yearly seasonal and occasional pandemics infections of great consequences to humans. While Influenza A viruses (IAV) circulate within several zoonotic hosts, providing the opportunity for pandemic, Influenza B viruses (IBV) primarily circulate in humans, and are not considered a pandemic threat. However, IBV are still major human pathogens responsible for seasonal epidemics of respiratory illness [1,2,3]. IBV infect humans of all ages but is responsible of more severe disease in young children, elderly, and immunocompromised populations [2,3,4,5,6,7,8,9]. Notably, epidemiological evidences indicates that the number of IBV cases has increased during the last years [10,11,12].

Similar to IAV, IBV are enveloped negative-sense, single-stranded segmented RNA viruses that belong to the *Orthomyxoviridae* family [13,14,15]. The eight IBV viral (v)RNAs encode for at least 11 proteins: PB2, PB1, and PA are the components of the viral RNA dependent RNA polymerase (RdRp) complex that, together with the viral nucleoprotein (NP) are responsible for viral genome replication and gene transcription [14,15,16]. Hemagglutinin (HA) and Neuraminidase (NA) are the two major glycoproteins and antigenic determinants in the surface of IBV; and responsible for viral entry and fusion (HA), and release from infected cells (NA) [14,15,16]. The matrix (M) segment encodes the matrix 1 (M1) and 2 (BM2) proteins, with the start codon of BM2 protein overlapping with the termination codon of the M1 protein [14,15,16]. IBV non-structural (NS) segment encodes, using an alternative splicing mechanism, the non-structural 1 (NS1) protein from the full-length transcript, and the nuclear export protein (NEP) from the pre-mRNA splicing [14,15,16]. IBV NS1 is mainly involved, similar to IAV NS1 [17,18,19,20,21,22,23,24,25,26,27], in counteracting type I interferon (IFN) responses [28,29,30,31,32,33]. NEP is mainly responsible for the trafficking of vRNAs from the nucleus to the cytoplasm at the latest stages of viral infection. One unique feature of IBV is the expression of the NB protein from the NA vRNA. NB is an ion channel protein essential for IBV replication [14,15,16,34]. Two separate IBV lineages (Yamagata and Victoria), which are evolutionary and antigenically distinct [35,36], originated in the 70s from a common influenza B/Lee/40 ancestor and, since then, have been co-circulating and re-assorting worldwide in humans [3,9,37,38,39] even within a given influenza epidemic [12,37,39,40].

IAV and IBV infections are most effectively prevented through vaccination [41,42]. However, and despite comprehensive and effective vaccination programs, the World Health Organization (WHO) estimates that the global disease burden from influenza results in 1 billion infections, 3–5 million cases of severe disease and between 300,000–500,000 deaths annually. United States (US) Food and Drug Administration (FDA)-approved vaccines for the prevention of seasonal IAV or IBV in humans include recombinant influenza vaccines (rIV), inactivated influenza vaccines (IIV), and live-attenuated influenza vaccines (LAIV). rIV and IIV are administered intramuscularly to induce protective humoral immunity by inducing the production of neutralizing antibodies that target the viral HA glycoprotein, and to a lesser extent NA, but are poor inducers of cellular immunity [43,44]. LAIV are given intranasally using a nasal spray and more closely mimic the natural route of influenza viral infection. Notably, LAIV have been shown to elicit broader cross-reactive humoral and cellular immune responses, providing better immunogenicity and protection efficacy than rIV and IIV, including protection against heterotypic viral infections [43,44,45,46,47]. For these reasons, LAIV are ideal to prevent and control influenza infections in humans as well as in other animals. Historically, influenza seasonal vaccines were made of trivalent formulations made of two IAV subtypes (H1N1 and H3N2) and one IBV lineage (Yamagata or Victoria) following WHO strain recommendations for each season [48]. However, since 2012, quadrivalent formulations of influenza vaccines containing representative strains of both IBV Victoria and Yamagata lineages, in addition to the two IAV H1N1 and H3N2 strains, have been approved and available to prevent seasonal influenza infections.

LAIV are generated using Master Donor Viruses (MDV) containing mutations that allow the virus to replicate at permissive low temperatures (33 °C), such as those in the upper respiratory tract (URT), but not at non-permissive high temperatures (37 °C) present in the lower respiratory tract (LRT). In the US, the MDV for IBV LAIV is B/Ann Arbor/1/1966. This MDV LAIV was generated by passaging wild-type (WT) B/Ann Arbor/1/1966 at progressive lower temperatures, resulting in a temperature sensitive (ts) variant that still replicate efficiently at low temperatures (cold-adapted, ca), but with an attenuated (att) phenotype in vivo [49,50,51]. The mutations responsible for the ts, ca and att phenotype of the IBV MDV used to generate LAIV have been mapped into the PB2 (S630R), PA (V341M), NP (V114A, P410H, and A509T), and M1 (H159Q and M183V) proteins [52,53,54,55]. The antigenic drift in influenza HA and NA needs for vaccines to be updated in a yearly based so that they antigenically match the influenza seasonal circulating strains [56]. IBV LAIV contain the six internal viral gene segments (PB2, PB1, PA, NP, M and NS) of the MDV B/Ann Arbor/1/1966 and the HA and NA viral genes of circulating seasonal IBV Yamagata and/or Victoria strains recommended by the WHO. Notably, the MDV of the US IAV (A/Ann Arbor/6/1960 H2N2) and IBV (B/Ann Arbor/1/1966). LAIV have been approved by the US FDA and used in the US since 2003 but the IAV and IBV MDV have not been updated. Notably, since the introduction of quadrivalent LAIV during the 2013-2014 influenza season [57,58], LAIV have been shown to have low efficacy in protecting against seasonal influenza viruses. Based on this low vaccine effectiveness data, the advisory committee on immunization practices (ACIP) concluded that a preference for the quadrivalent LAIV over the IIV was no longer warranted. The cause(s) of reduced effectiveness of quadrivalent LAIV are still unknown but this could be, at least in part, because of the lack of sequence similarities in the internal viral proteins of the MDV LAIV and circulating seasonal strains. Therefore, MDV based on more recent IAV and IBV strains may represents a better option for their use as MDV for the development of LAIV because on their most conserved amino acid sequence with seasonal strains.

We and others have previously shown that introducing the mutations of the US IAV MDV A/Ann Arbor/6/1960 H2N2 LAIV (PB2 N265S and PB1 K391E, D581G, and A661T) [59,60] resulted in the transfer of the ts, ca and att phenotype to other IAV strains, including A/Puerto Rico/8/1934 H1N1 (PR8) [61,62], pandemic A/California/04/2009 H1N1 (pH1N1) [63], A/canine/NY/dog23/2009 H3N8 [64,65,66], and A/equine/Ohio/1/2003 H3N8 [67,68] viruses. Likewise, mutations responsible for the ts, ca and att phenotype of the Russian IAV MDV A/Leningrad/134/17/1957 H2N2 LAIV (PB2 V478L; PB1 K265N and V591I; and NEP M100I) [69] resulted in the same ts, ca and att phenotype when introduced in PR8 [62] and pH1N1 [63] IAV. Notably, a single intranasal dose vaccination with PR8 [62] or pH1N1 [63] containing these ts, ca and att mutations were able to protect against lethal challenge with WT forms of these viruses, demonstrating the feasibility of using these new ts, ca, and att PR8 [62] or pH1N1 [63] as MDV LAIV for IAV. Notably, we have previously shown how the mutations of the US IAV MDV LAIV A/Ann Arbor/6/1960 H2N2 PB1, together with an HA epitope tag in the C terminus of IBV PB1, were able to confer a ts phenotype in vitro and an att phenotype in vivo to B/Brisbane/60/2008, while protecting against challenge with IBV [70].

In this manuscript we evaluated whether the ts, ca and att mutations present in IBV MDV LAIV B/Ann Arbor/1/1966 would be able to confer the same phenotype to a most recent B/Brisbane/60/2008 strain and, importantly, if this new IBV MDV LAIV will be able to protect, using a single intranasal vaccination, against challenge with WT B/Brisbane/60/2008 in a mouse model of infection. Using reverse genetics approaches, we generated a recombinant B/Brisbane/60/2008 containing the mutations in PB2 (S630R), PA (V341M), NP (P410H and A509T; V114 was already present in B/Brisbane/60/2008 WT), and M1 (H159Q and M183V) previously identified to be responsible of the ts, ca and att phenotype of B/Ann Arbor/1/1966 MDV LAIV to generate a B/Brisbane/60/2008 MDV LAIV. Our results demonstrate that B/Brisbane/60/2008 containing B/Ann Arbor/1/1966 MDV LAIV mutations has a similar ts and ca phenotype in vitro and att phenotype in vivo. Importantly, a single intranasal vaccination with our novel IBV MDV LAIV was able to protect against a challenge with WT B/Brisbane/60/2008. Altogether, these results demonstrate the feasibility of updating the current IBV MDV for the development of IBV LAIV based on the use of a more recent B/Brisbane/60/2008 strain.

## 2. Materials and Methods

### 2.1. Cells and Viruses

Human embryonic kidney 293T (American Type Culture Collection, ATCC CRL-11268) and Madin-Darby Canine Kidney, MDCK (ATCC CCL-34) cells were grown and maintained in Dulbecco Modified Eagle Medium (DMEM) and supplemented with 10% fetal bovine serum (FBS) and 1% PSG (Penicillin, Streptomycin, l-glutamine) in a 5% CO_2_ incubator at 37 °C. Influenza B/Brisbane/60/2008 was generated using plasmid-based reverse genetics, as previously described [16,71,72]. Virus stocks were generated and titrated in MDCK cells as previously defined [16,71]. Influenza B/Brisbane /60/2008 expressing the mCherry fluorescent protein has been previously described [73].

### 2.2. Plasmids

Plasmids for the generation of recombinant B/Brisbane/60/2008 WT have been previously described [71]. Mutations responsible for the ts, ca, and att phenoytype of B/AnnArbor/01/1966 MDV LAIV (PB2 S630R, PA V431M, NP P410H and A509T, and M1 H159Q and M183V) were incorporated into pDP plasmids containing the B/Brisbane/60/2008 WT viral genes [70]. Plamids were Sanger sequenced (ACGT Inc.) to confirm the presence of the ts, ca and att B/AnnArbor/01/1966 MDV LAIV mutations in the B/Brisbane/60//2008 MDV LAIV PB2, PA, NP and M plasmids.

### 2.3. Minigenome Assays

Human 293T cells (12-well plate format, 5 × 10^5^ cells/well, tripllicates) were transiently transfected, using Lipofectamine 2000, with 500 ng of WT or LAIV pDP-PB2, pDP-PB1, pDP-PA and pDP-NP B/Brisbane/60/2008 plasmids, together with 500 ng of human polymerase I (pPolI) plasmids expressing GFP (pPolI-IBV_GFP) and Firefly luciferase (pPolI-IBV_FLuc), and 50 ng of a Simian Virus 40 (SV40) Renilla luciferase (Rluc) expression plasmid (pSV40_RLuc). At 48 h post-transfection, GFP expression was determined under a fluorescent microscope. FLuc and RLuc expression levels were determined at the same time post-transfection using a dual luciferase kit (Promega). The activity of B/Brisbane/60/2008 MDV LAIV polymerase activity was normalized to that of B/Brisbane/60/2008 WT polymerase, which was set to 100%.

### 2.4. Virus Rescue

Viral rescues were conducted as previously described [71]. Briefly, MDCK and 293T cells (6-welll plate format, 10^6^ cells/well, triplicates) were transiently co-transfected, using Lipofectamine 2000, with pDP plasmids encoding the 8 viral gene segments of B/Brisbane/60/2008 WT or MDV LAIV. At 8 h, transfection media was replaced with post-infection media and cells were incubated at 33 °C. After 3 days, tissue culture supernatants were collected and used to infect fresh monolayers of MDCK cells (6-welll plate format, 10^6^ cells/well, triplicates). Four days after infection, presence of B/Brisbane/60/2008 WT or MDV LAIV was determined by HA assay.

### 2.5. Virus Growth Kinetics

Confluent monolayers of MDCK cells (12-well plates, 5 × 10^5^ cells/well, triplicates) were infected (MOI of 0.01) for 1 h at room temperature. After 1 h virus absorption, virus was removed and replaced with post-infection media. Plates were incubated in 5% CO_2_ incubators at either 33 °C or 37 °C. At the indicated h post-infection, tissue culture supernatants were collected and viral titers were determined by immunofocus assay (fluorescent forming units [FFU]/mL). Briefly, confluent monolayers of MDCK cells (96-well plates, 10^4^ cells/cell, triplicates) were infected with 50 μL of virus from each time point (in triplicate) using 10-fold serial dilutions. Plates were placed at 33 °C for 12 h. After viral infection, cells were fixed and permeabilized with 4% formaldehyde and 0.5% Triton-X100 in 1× PBS. A primary monoclonal antibody against IBV NP (B017, AbCam Cat. Ab20711-100) was added to each well and incubated at 37 °C for 90 min. After 3× washes with 1× PBS, a secondary FITC-conjugated polyclonal rabbit anti-mouse antibody (Abcam Cat. ab6724) was added to each well and incubated at 37 °C for 45 min. After 3× washes with 1× PBS, plates were observed under an Olympus fluorescent microscope to quantify positive stained cells.

### 2.6. Plaque Assays

Confluent monolayers of MDCK cells (6-well plate format, 10^6^ cells/well) were infected with B/Brisbane/60/2008 WT or MDV LAIV for 1 h at room temperature. After 1 h infection, cells were overlaid with DMEM/F-12 containing 2% Oxoid agar and incubated at 33 °C or 37 °C in 5% CO_2_. At 72 h post-infection, cells were fixed overnight with 2.5% formalin (in 1× PBS) at 4 °C. After overnight incubaton, agar overlays were removed and cells were fixed and permeabilized using 4% formaldehyde with 0.01% Triton-X100 in 1× PBS at room temperature for 1 h. Immunostaining of viral plaques was performed using the IBV anti-NP monoclonal antibody B017 and vector kits (Vectastain ABC kit and DAB HRP Substrate Kit: Vector), according to manufacturer’s specifications.

### 2.7. Animal Experiments

Six-week-old, female C57BL/6J mice were obtained from the National Cancer Institute (NCI) and maintain in the University of Rochester Animal Care Facilities under protocols outlined by the University Committee of Animal Reseources (UCAR). Mice were anesthetized through intraperitoneal injection of Avertin (240 mg/kg of body weight) and subsequently infected intranasally with 10^6^ FFU of either B/Brisbane/60/2008 WT or MDV LAIV [74]. An additional cohort of mice (*n* = 6) was anesthetized as described above and treated with sterile 1× PBS as a negative control [74]. To determine viral titers in vivo, lung and nasal turbinates were harvest at days 2 (*n* = 3) and 4 (*n* = 3) post-infection [74]. Harvested tissues were homogenized and viral titers were determined by immunofocus assay (FFU/mL) [74]. Serum from infected mice were collected through submandibular bleeds 14 days post-infection [74]. On day 28 post-infection, mice were challenged with B/Brisbane/60/2008 WT (10^6^ FFU). Mice were euthanized at day 2 and 4 post-challenge. Lungs were harvested and viral titers determined as described above [74].

### 2.8. Enzyme-Linked Immunosorbent Assay (ELISA)

To determine total IBV specific antibodies, serum collected at day 14 post-infection from infected mice was used in ELISA assays. Briefly, 96-well plates were coated at 250 ng/well with either B/Ohio/01/2005 (BEI Resources, NR-19243) or B/Malaysia/2506/2004 (BEI Resources, NR-51162) recombinant HA proteins and placed at 4 °C overnight. Plates were then blocked with 3% BSA in 1× PBS for 1 h at 37 °C. Serum diluted in 0.5% BSA in 1× PBS was then added to the plate using two-fold dilutions, starting 1:100 dilution, and incubated at room temperature for 2 h. Plates were washed 3× with ELISA wash buffer (1× PBS + 0.05% Tween) and then 50 μL of a horseradish peroxidase-conjugated goat anti-mouse IgG secondary antibody (1:2000; Southern Biotech) was added to each well for 1 h at 37 °C. Reactions were developed with tetramethylbenzidine (TMB) substrate (BioLegend) for 10 min at room temperature. Reaction was stopped with 2N H_2_SO_4_ and read at 450 nm using the Vmax kinetic microplate reader (Molecular Devices).

### 2.9. Hemagglutination Inhibition (HAI) Assay

Serum from mice infected with either B/Brisbane/60/2008 WT or MDV LAIV at day 14 post-infection were RDE (receptor-destroying enzyme) treated overnight at 37 °C and then placed at 56 °C for 30 min for heat inactivation. To determine the presence of neutralizing antibodies, heat inactivated serum were diluted in 96-well V-bottom plates and incubated with 4 hemagglutinating units (HAU) of B/Brisbane/60/2008 WT and left at room temperature for 60 min. Then, 0.5% turkey red blood cells were added to each well and left on ice for 45 min. HAI antibody titers were calculated based on the last well in which hemagglutination did not occur, with the riciprocal of the corresponding dilution being the hemagglutination titer.

### 2.10. Microneutralization Assays

To evaluate neutralizing antibodies against B/Brisbane/60/2008 WT elicited by infection with either WT or MDV LAIV B/Brisbane/60/2008, serum from infected mice were collected at day 14 post-infection, treated as outlined above, and serially diluted in 96-well plates. B/Brisbane/60/2008 expressing mCherry (100 FFU/well) [73,75] was added to the 96-well plate and incubated at room temperature for 1 h Next, 50 μL of the serum/virus mixture was added to confluent monolayers of MDCK cells in 96-well plate replicas and left at room temperature for 1 h to allow viral infection. After 1 h viral infection, serum/virus media was replaced with post-infection media. At 48 h a fluorescent microplate reader was used to quantify mCherry expression [73,75,76]. Neutralizing capacity was determined by normalizing to mCherry expression in the absence of sera [73,75,76].

## 3. Results

### 3.1. The Mutations of B/Ann Arbor/1/1966 MDV LAIV Confer a ts and ca Phenotype to B/Brisbane/60/2008

The first step towards identifying a new IBV MDV LAIV was to identify the ideal viral candidate. B/Brisbane/60/2008 was selected as the potential MDV LAIV candidate because this Victoria lineage IBV circulated more recently (2008) within the human population and because its internal genes have increased percent identity and sequence homology with contemporary IBV strains circulating in humans, relatively to the current B/Ann Arbor/1/1966 MDV LAIV (Table 1, supplementary material, and data not shown).

**Figure 1:**
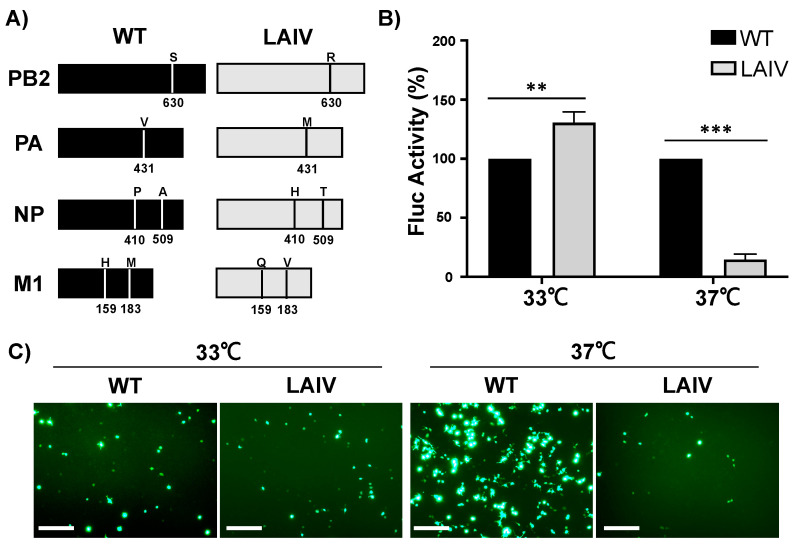
Influenza B/Ann Arbor/1/1966 MDV LAIV mutations confer a ts and ca phenotype to the polymerase and NP of B/Brisbane/60/2008. (**A**) Schematic representation of the PB2, PA, NP, and M1 viral proteins of B/Brisbane/60/2008 WT (left) and MDV LAIV (right): Six of the seven mutations responsible for the ts, ca and att phenotype of B/Ann Arbor/1/1966 MDV LAIV (NP mutation V114A was already present in B/Brisbane/60/2008 WT) were introduced into the PB2, PA, NP and M1 proteins to generate the B/Brisbane/60/2008 MDV LAIV. (**B**,**C**) Minigenome activity: Human 293T cells were transfected with 500 ng of pDP expression plasmids encoding the four proteins required for IBV replication and transcription (pDP-PB2, pDP-PB1, pDP-PA and pDP-NP) together with minigenome plasmids encoding Fluc or GFP flanked by IBV non-coding regions under the control of the human polymerase I promoter, and a SV40-driven polymerase II promoter Renilla luciferase (Rluc) expression plasmid to normalize transfection efficiencies. After 6 h transfection, cells were placed at either 33 or 37 °C. Viral genome replication and gene transcription were determined at 48 h post-transfection by Fluc (B) and GFP (C) expression. WT FLuc/Rluc expression level was normalized to 100% and LAIV Fluc/Rluc expression calculated relative to WT. Data is represented as the mean and SD. Student t test was performed to determine *p* values. **, *p* < 0.01; ***, *p* < 0.001. Representative images of GFP expression by WT or MDV LAIV B/Brisbane/60/2008 polymerases and NPs at the indicated temperatures are shown. Scale bars = 100 µM.

In order to generate a virus that only replicate efficiently at 33 °C, but not at 37 °C, for its safe implementation as a MDV for the development of IBV LAIV, we first evaluated if the mutations responsible for the ts, ca, and att phenotype of B/Ann Arbor/1/1966 MDV LAIV were able to confer a similar phenotype to B/Brisbane/60/2008 (Figure 1). To that end, 6 of the 7 mutations previously described to be responsible of the ts, ca, and att phenotype of the B/Ann Arbor/1/1966 MDV LAIV [52,53,54,55] were introduced, using site directed mutagenesis, in the PB2 (S630R), PA (V431M), NP (P410H and A509T) and M1 (H159Q and M183V) proteins of B/Brisbane/60/2008 (Figure 1A). B/Ann Arbor/1/1966 MDV LAIV NP mutation V114A was already present in B/Brisbane/60/2008 WT. To evaluate if the introduced mutations inferred the anticipated ts and ca phenotype, we first conducted minigenome assays at 33 °C and 37 °C (Figure 1B,C). At 33 °C, Firefly luciferase (Fluc) expression levels in cells transfected with the polymerase PB2, PA, and NP of B/Brisbane/60/2008 MDV LAIV were significantly elevated relative to B/Brisbane/60/2008 WT (Figure 1B), indicating that these mutations have induced a ca phenotype to the polymerase of B/Brisbane/60/2008. Importantly, B/Brisbane/60/2008 MDV LAIV polymerase and NP complex underperformed at 37 °C, with Fluc expression levels significantly reduced relative to those of B/Brisbane/60/2008 WT, suggesting that these mutations also conferred a ts phenotype to B/Brisbane/60/2008 polymerase and NP (Figure 1B). Minigenome GFP expression levels were also evaluated to determine the polymerase PB2, PA, and NP activities of both WT and MDV LAIV B/Brisbane/60/2008 (Figure 1C). At 33 °C, GFP expression levels were comparable between WT and MDV LAIV B/Brisbane/60/2008. Conversely, at 37 °C, GFP expression levels by B/Brisbane/60/2008LAIV were drastically reduced compared to those of B/Brisbane/60/2008 WT (Figure 1C). These results indicate that the mutations responsible for the ts and ca phenotype of B/Ann Arbor/1/1966 MDV LAIV were able to confer a similar phenotype to the polymerase PB2, PA, and NP of B/Brisbane/60/2008.

### 3.2. Influenza B/Brisbane/60/2008 WT Replication

**Figure 2:**
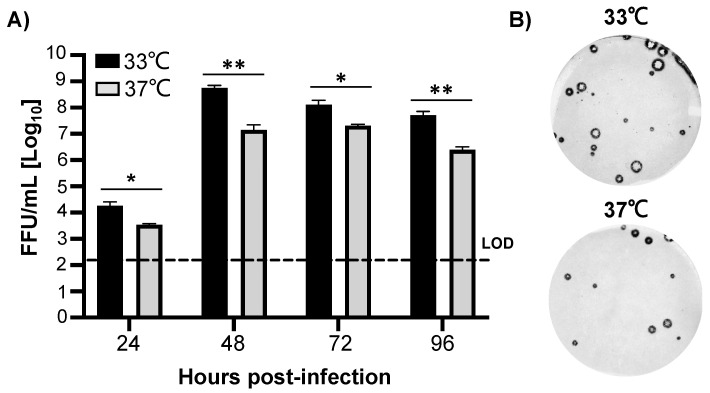
Influenza B/Brisbane/60/2008 WT replication at different temperatures. (**A**) Viral growth kinetics: Confluent monolayers of MDCK cells were infected (MOI 0.01 FFU/cell) with B/Brisbane/60/2008 WT. After 1 h infection cells were incubated at 33 °C (black) or 37 °C (gray). Tissue culture supernatants were collected at 24, 48, 72 and 96 h post-infection. Viral titrations were determined using an immunofocus assay (fluorescent forming units, FFU/mL) with a mouse monoclonal antibody against IBV NP. Data is represented as the mean and SD. Dotted line indicates the limit of detection (LOD) of the assay (200 FFU). Student t test was performed to determine *p* values. *, *p* < 0.05; **, *p* < 0.01. (**B**) Plaque assays: Confluent monolayers of MDCK cells were infected with B/Brisbane/60/2008 WT. After 1 h infection, cells were overlaid with media containing agar and incubated at 33 °C (top) or 37 °C (bottom). At 72 h post-infection viral plaques were immunostained with an anti-IBV NP mouse monoclonal antibody.

To determine if B/Brisbane/60/2008 WT would be a suitable MDV LAIV candidate, we first evaluate its ability to replicate at 33 °C and 37 °C in MDCK cells (Figure 2). B/Brisbane/60/2008 WT was able to efficiently replicate at both 33 °C and 37 °C, with viral titers reaching 10^8^ FFU/mL and 10^7^ FFU/mL, respectively, at 48 h post-infection (Figure 2A). Next, we evaluated the ability of B/Brisbane/60/2008 WT to replicate at the same temperatures by plaque assay (Figure 2B). We also conducted similar viral growth kinetics and plaque assays at 39 °C but we were not able to detect B/Brisbane/60/2008 WT in the tissue culture supernatants of infected cells, or viral plaques, respectively (data not shown).

### 3.3. Generation and Characterization of B/Brisbane/60/2008 MDV for the Development of IBV LAIV

**Figure 3:**
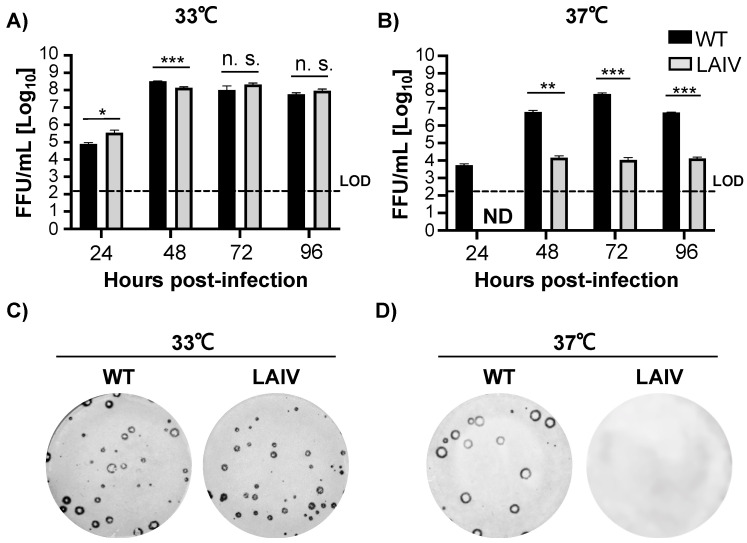
B/Brisbane/60/2008 WT and MDV LAIV replication at different temperatures. (**A**,**B**) Viral growth kinetics: MDCK cells were infected (MOI of 0.01 FFU/cell) with either B/Brisbane/60/2008 WT (black) or MDV LAIV (grey) and incubated at 33 °C (**A**) or 37 °C (**B**). Tissue culture supernatants from infected cells were collected at 24, 48, 72 and 96 h post-infection and virus titers were determined by immunofocus assay (FFU/mL) with a mouse monoclonal antibody against IBV NP. Dotted lines indicate the LOD of the assay (200 FFU). ND = Not detected. Data is represented as the mean and SD. Student t test was performed to determine *p* values. *, *p* < 0.05; **, *p* < 0.01; ***, *p* < 0.001; n.s.: not significant. (**C**,**D**) Plaque assays: MDCK cells were infected with either B/Brisbane/60/2008 WT or MDV LAIV and after 1 h infection, cells were overlaid with media containing agar and incubated at 33 °C (**C**) or 37 °C (**D**). At 72 h post-infection viral plaques were immunostained with an anti-IBV NP mouse monoclonal antibody.

Next, we used our previously described plasmid-based reverse genetics techniques [16,71,72] to generate a recombinant B/Brisbane/60/2008 MDV LAIV and evaluate viral replication and plaque phenotype in MDCK cells at 33 °C and 37 °C, and compared them to those of B/Brisbane/60/2008 WT (Figure 3). At 33 °C, B/Brisbane/60/2008 MDV LAIV replicated (Figure 3A) and formed similar viral plaque sizes (Figure 3B) than those of B/Brisbane/60/2008 WT. At 37 °C, however, viral titers of B/Brisbane/60/2008 MDV LAIV were significantly reduced compared to those of B/Brisbane/60/2008 WT at all timepoints (Figure 3B). Likewise, while B/Brisbane/60/2008 WT was able to plaque at 37 °C, we could not detect any viral plaques with B/Brisbane/60/2008 MDV LAIV at 37 °C, (Figure 3D). These results further support that the ts and ca mutations of B/Ann Arbor/1/1966 MDV LAIV were able to confer a similar phenotype to B/Brisbane/60/2008 and that viral replication of B/Brisbane/60/2008 MDV LAIV is restricted to permissive (33 °C) temperatures.

### 3.4. Safety of B/Brisbane/60/2008 MDV LAIV

**Figure 4:**
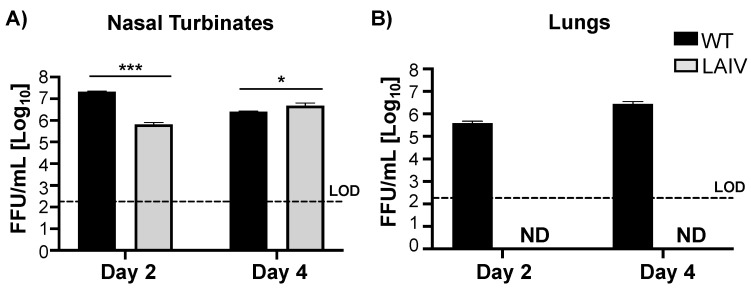
B/Brisbane/60/2008 MDV LAIV replication in vivo: C57BL/6J 6-weeks-old female mice (*n* = 3) were infected intranasally with 10^6^ FFU of B/Brisbane/60/2008 WT (black) or MDV LAIV (grey). Nasal turbinates (**A**) and lungs (**B**) were collected at days 2 and day 4 post-infection and viral titers were determined by immunofocus assay (FFU/mL). Dotted lines indicate the LOD of the assay (200 FFU). Data is represented as the mean and SD. Student t test was performed to determine *p* values. *, *p* < 0.05; ***, *p* < 0.001. ND = Not detected.

In order to implement B/Brisbane/60/2008 MDV as a safe intranasal LAIV, we first confirmed that replication of B/Brisbane/60/2008 MDV LAIV was restricted to the lower temperatures of the upper respiratory track. To that end, 6-week old, female C57BL/6J mice were infected with 10^6^ FFU of B/Brisbane/60/2008 MDV LAIV or WT and viral replication in the upper (nasal turbinate) and lower (lungs) respiratory track were evaluated at days 2 and 4 post-infection (Figure 4). We were able to detect B/Brisbane/60/2008 WT in both the nasal turbinate (Figure 4A) and the lungs (Figure 4B) of infected animals. Viral titers of B/Brisbane/60/2008 MDV LAIV in the nasal turbinate were slighted, but significantly, reduced (~1 log) to those of B/Brisbane/60/2008 WT at day 2 post-infection (Figure 4A). However, at day 4 post-infection, viral titers in the nasal turbinate of mice infected with B/Brisbane/60/2008 MDV LAIV were significantly higher than those of B/Brisbane/60/2008 WT (Figure 4A). Notably, we were not able to detect B/Brisbane/60/2008 MDV LAIV in the lungs of infected mice at days 2 or 4 post-infection (Figure 4B). These results suggest that B/Brisbane/60/2008 MDV LAIV is able to replicate efficiently in the URT but is completely impaired in viral replication in the LRT of infected mice, important for its implementation as a safe LAIV.

### 3.5. Immunogenicity of B/Brisbane/60/2008 MDV LAIV

**Figure 5:**
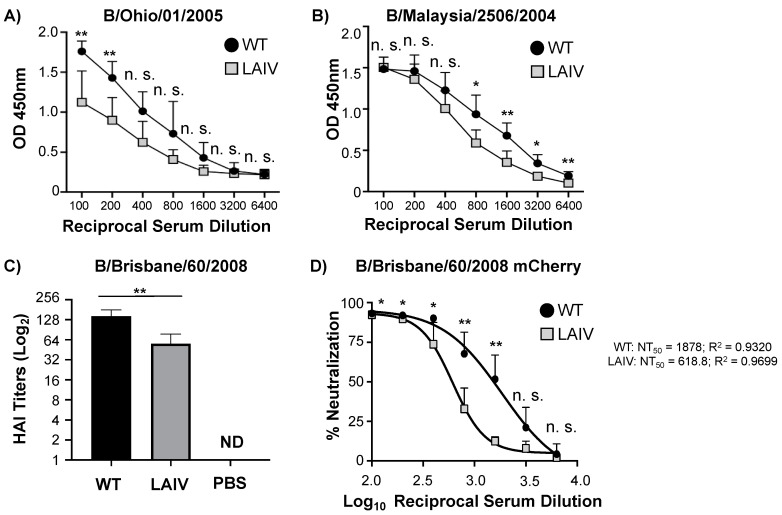
B/Brisbane/60/2008 MDV LAIV immunogenicity: C57BL/6J 6-weeks-old female mice (*n* = 6) were intranasally infected with 10^6^ FFU of B/Brisbane/60/2008 WT (black) or MDV LAIV (grey). Serum was collected at 14 days post-infection and RDE treated/heat inactivated. (**A**,**B**) ELISA: Total IgG Ab responses were determined by ELISA with purified HA protein from either B/Ohio/1/2005 (**A**) or B/Malaysia/2506/2004 (**B**) Victoria lineage IBV. OD, Optical Density. (**C**,**D**) HAI and microneutralization assays: Neutralizing antibodies against B/Brisbane/60/2008 were determined by HAI (**C**) and fluorescent-based microneutralization (**D**) assays. Data is represented as the mean and SD. Student t test was performed to determine *p* values. *, *p* < 0.05; **, *p* < 0.01; n.s.: not significant. ND = Not detected.

Next, we evaluated the ability of B/Brisbane/60/2008 MDV LAIV to induce antibody-mediated immune responses. To determine the magnitude and breadth of the antibody-mediated immune response elicited by B/Brisbane/60/2008 MDV LAIV, 6-week old, female C57BL/6J mice were infected intranasally with 10^6^ FFU of either B/Brisbane/60/2008 WT or MDV LAIV (Figure 5). Sera from animals were collected 14 days post-infection and assayed by enzyme-linked immunosorbent assay (ELISA) to determine total IgG responses against purified HA of two Victoria lineage IBV (B/Ohio/01/2005 and B/Malaysia/2506/2004) strains. Total IgG responses against both IBV HA proteins were detectable in mice infected with B/Brisbane/60/2008 MDV LAIV at levels slightly reduced, but statistically significant, to those elicited by B/Brisbane/60/2008 WT infection (Figure 5A,B). Hemagglutinin inhibition (HAI) assays were next conducted to evaluate the presence of neutralizing antibodies against B/Brisbane/60/2008 (Figure 5C). Higher HAI antibody titers against B/Brisbane/60/2008 were detected in the serum of mice infected with B/Brisbane/60/2008 WT as compared to those of B/Brisbane/60/2008 MDV LAIV. These results were further confirmed using a fluorescent-based microneutralization assays based on the use of mCherry-expressing B/Brisbane/60/2008 WT (Figure 5D) [73,75]. Neutralizing antibodies from mice infected with B/Brisbane/60/2008 WT were statistically higher than those obtained with B/Brisbane/60/2008 MDV LAIV. These results demonstrate the ability of B/Brisbane/60/2008 MDV LAIV to elicit total, HAI and neutralizing antibody responses against B/Brisbane/60/2008 WT, although to a less extend than those induced by B/Brisbane/60/2008 WT infection.

### 3.6. B/Brisbane/60/2008 MDV LAIV Protection Efficacy

**Figure 6:**
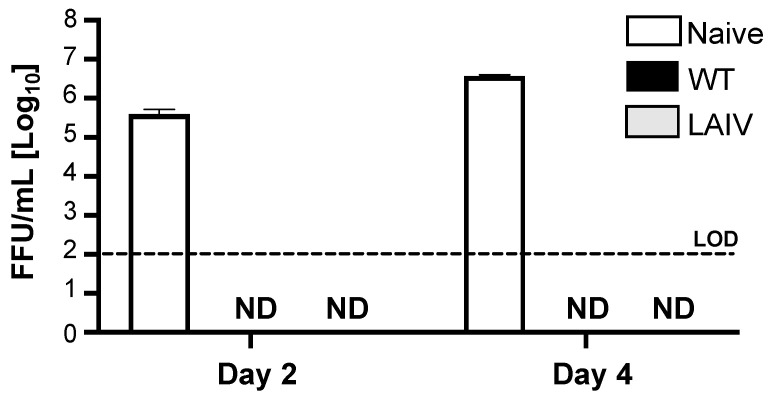
Protective efficacy of B/Brisbane/60/2008 MDV LAIV: C57BL/6J 6-weeks old female mice (*n* = 6) were infected intranasally with 10^6^ FFU of either B/Brisbane/60/2008 WT (black) or MDV LAIV (gray), or mock vaccinated (white). On day 28 post-infection, mice were challenged with 10^6^ FFU of B/Brisbane/60/2008 WT. At days 2 (*n* = 3) and day 4 (*n* = 3) post-challenge, mice lungs were collected and viral titers were determined by immunofocus assay (FFU/mL). Dotted line indicates the LOD of the assay (200 FFU/mL). ND= Not detected.

Our previous results demonstrate that B/Brisbane/60/2008 MDV LAIV is capable of inducing neutralizing antibody responses against B/Brisbane/60/2008 but the true hallmark of an effective neutralizing humoral responses to protect against challenge needed to be evaluated. To demonstrate that immunization with B/Brisbane/60/2008 MDV LAIV protects against B/Brisbane/60/2008 WT, C57BL/6J mice were vaccinated intranasally with 10^6^ FFU of either B/Brisbane/60/2008 WT or MDV LAIV (Figure 6). A third cohort of mice, referred to as naïve, were mock (PBS)-vaccinated. After 28 days vaccination, the three groups of mice were challenged intranasally with 10^6^ FFU of B/Brisbane/60/2008 WT and lungs from challenged mice were harvested at days 2 and 4 post-infection to evaluate B/Brisbane/60/2008 WT viral titers. While we were able to detect B/Brisbane/60/2008 WT in the lungs of naïve-vaccinated mice, we were not able to detect B/Brisbane/60/2008 WT in the lungs of WT or MDV LAIV B/Brisbane/60/2008 vaccinated groups (Figure 6). This results demonstrate that B/Brisbane/60/2008 MDV LAIV is able to protect against a challenge with B/Brisbane/60/2008 WT.

## 4. Discussion

IBV are no less important than their IAV counterparts as etiological agents of seasonal influenza epidemics. Although IBV contribute less to epidemics than IAV H3N2 strains, they contribute more than IAV H1N1 strains [10] and are the predominant circulating influenza viruses approximately once every 2–4 years [2,3,4,77]. IBV are associated with outbreaks of illness in adults and the elderly [4,5,6,7,8,10] and with an excess morbidity and mortality in the pediatric population [2,3,38]. During the last few decades, IBV has been the cause of several outbreaks, including acute respiratory illness in cruises [78], schools [79,80], and the military [81]; non-respiratory clinical outcomes [2,8,82,83,84,85,86] and secondary bacterial pneumonia infections [86,87,88]. IBV public health concerns are aggravated by their efficient transmission [89] and the lack of anti-viral effectiveness [90,91,92,93,94,95,96,97,98,99]. The relevance of IBV in human health has been demonstrated by the recently inclusion of appropriate strains of the two circulating lineages (Yamagata and Victoria) of IBV in the yearly influenza seasonal vaccine formulations to reduce annual cases, hospitalizations, and deaths.

Here, we describe the development of a new IBV MDV LAIV candidate in the backbone of a recent B/Brisbane/60/2008 strain. Our results demonstrate how the ts, ca, and att mutations of the current US FDA-approved MDV LAIV B/Ann Arbor/1/1966 into B/Brisbane/60/2008 PB2, PB1, NP and M resulted in a ts and ca phenotype in vitro (Figure 1) able to replicate to high titers at permissive temperatures, important for vaccine production (Figure 3). Using a mouse model we also demonstrate that the new B/Brisbane/60/2008 MDV LAIV is only able to replicate in the URT but not in the LRT of infected mice (Figure 4), important for its safe implementation as a MDV to generate IBV LAIV. Notably, a single intranasal immunization with this new B/Brisbane/60/2008 MDV LAIV was able to induce robust humoral immune responses, including HAI and neutralizing antibodies, against IBV (Figure 5) and complete protection against challenge with B/Brisbane/60/2008 WT (Figure 6). Based on these results, we hypothesize that this new MDV based on a more recent IBV seasonal strain could be used to generate LAIV containing the internal genes of the B/Brisbane/60/2008 MDV LAIV and the HA and NA glycoproteins of contemporary Yamagata and Victoria strains for the more efficient treatment of IBV infections in humans.

Since the last few years, there is a global interest in developing universal vaccine approaches that require fewer updates and provide long-lasting immunity for the treatment of influenza infections [100,101,102,103,104]. Since their initial development, LAIV are based on the use of the internal genes of MDV that contain mutations able to replicate efficiently at low temperatures (ts, ca) with a safety profile (att) and the HA and NA viral glycoproteins of seasonal circulating IAV or IBV. These include the US MDV A/Ann Arbor/6/60 H2N2 (IAV) and B/Ann Arbor/1/1966 (IBV). The ts, ca, and att A/Ann Arbor/6/60 H2N2 and B/Ann Arbor/1/1966 have been licensed for human use since 2003 and they have been used as MDV for the generation of both seasonal and potentially pandemic, in the case of IAV, human LAIV by creating reassortant viruses containing the six internal vRNA segments PB2, PB1, PA, NP, M and NS of the IAV or IBV MDV, and the viral glycoprotein-encoding vRNAs HA and NA from viruses that antigenically match the strains predicted to circulate in the upcoming influenza season or potentially pandemic strains. However, since their implementation, these IAV or IBV MDV have not been updated.

The reason(s) why LAIV had low effectiveness since the 2013-2014 season remains unknown. One of the potential explanations for the lack of effectiveness of LAIV is the use of same IAV and IBV MDV backbones. If this is the case, updating the MDV of IAV (A/Ann Arbor/6/60 H2N2) and IBV (B/Ann Arbor/1/1966) to different and more contemporary strains backbones, could solve this problem and provide with more effective LAIV for the treatment of both IAV and IBV infections. We have previously shown how the ts, ca, and att mutations of the US MDV A/Ann Arbor/6/60 H2N2 were able to confer a similar phenotype to other IAV strains, including PR8 [61,62] and pH1N1 [63], A/canine/NY/dog23/2009 H3N8 [64,65,66], and A/equine/Ohio/1/2003 H3N8 [67,68] viruses. Likewise, we have also shown that the mutations responsible for the Russian MDV A/Leningrad/17/1957 H2N2 also conferred a similar ts, ca and att phenotype to PR8 [62] and pH1N1 [63] IAV. Moreover, we have shown how a LAIV elicits enhanced protection when the internal genes of the vaccine match those of the challenge virus [63]. Here, we demonstrate that the same experimental approach can be used for the development of an updated IBV MDV LAIV. Another reason is that vaccine strains must represent viruses currently in circulation. In this regard, we have demonstrated how incorporation of the ts, ca and att mutations of the IAV and IBV MDV in more recent strains represent a reasonable option to implement alternative and more protective LAIV. This could prevent potential outbreaks based on vaccine strains not matching circulating viruses. Importantly, these strategies will still use the same production and manufacturing currently used to generate LAIV and, therefore, will not need adaptation to what it is currently in use to develop LAIV. Moreover, the new IBV MDV LAIV is based on mutations that have been previously described to be responsible for the ts, ca and att phenotype of B/Ann Arbor/1/1966 MDV LAIV, which have a proven history of safety and stability. In addition, since this new IBV MDV LAIV based on the use of B/Brisbane/60/2008 was generated using plasmid-based reverse genetics approaches [16,71,72], it would be possible to easily update IBV LAIV by simply combining the internal viral genes of B/Brisbane/60/2008 MDV LAIV and the HA and NA of Yamagata or Victoria lineage new seasonal strains for the rapid development of updated IBV LAIV. Based on these multiple advantages over current approaches, our novel IBV MDV LAIV platform represents an excellent option for its implementation to prevent and control IBV infections. Future studies aimed to evaluate safety, immunogenicity and protection efficacy in a more relevant animal model would be needed for the application of this new MDV LAIV for the treatment of IBV infections in humans.

## Figures and Tables

**Table 1:** Nucleotide and amino acid sequence comparison of B/Ann Arbor/1/1966 and B/Brisbane/60/2008.

Viral Gene Protein	B/Ann Arbor/1/1966 Gene	B/Brisbane/60/2008 Gene	B/Ann Arbor/1/1966 Protein	B/Brisbane /60/2008 Protein	Amino Acid Similarity
PB2	(M20168.1) ^1^ 2396 nt	(CY115156.1) ^1^ 2358 nt	(AAA47771.1) ^2^ 770 aa	(AFH57919.1) ^2^ 770 aa	97.8%
PB1	(M20170.1) ^1^ 2369 nt	(CY115157.1) ^1^ 2334 nt	(AAA43770.1) ^2^ 752 aa	(AFH57918.1) ^2^ 752 aa	98.4%
PA	(M20170.1) ^1^ 2308 nt	(CY115156.1) ^1^ 2245 nt	(AAA43766.1) ^2^ 726 aa	(AFH57917.1) ^2^ 726 aa	98.0%
NP	(M20174.1) ^1^ 1842 nt	(CY115154.1) ^1^ 1750 nt	(AAA66419.1) ^2^ 560 aa	(AFH57914.1) ^2^ 560 aa	97.5%
M1	(M20176.1) ^1^ 770 nt	(CY115152.1) ^1^ 748 nt	(AAA66416.1) ^2^ 248 aa	(AFH57910.1) ^2^ 248 aa	100%
M2	(M20176.1) ^1^ 418 nt	(CY115152.1) ^1^ 399 nt	(AAA66416.1) ^2^ 109 aa	(AFH57910.1) ^2^ 109 aa	90.8%
NSI	(M20225.1) ^1^ 732 nt	(CY115155.1) ^1^ 722 nt	(AAA43759.1) ^2^ 281 aa	(AFH57915.1) ^2^ 282 aa	95.0%
NEP	(M20225.1) ^1^ 399 nt	(CY115155.1) ^1^ 380 nt	(AAA43759.1) ^2^ 122 aa	(AFH57916.1) ^2^ 123 aa	96.7%

^1^ Nucleotide sequence accession number in pubmed. ^2^ Amino acid sequence accession number in pubmed.

## Data Availability

Not applicable.

## Supplementary Materials

The following are available online at https://www.mdpi.com/article/10.3390/v13071278/s1, The amino acid sequence alignment of B/Ann Arbor/1/1966 and B/Brisbane/60/2008 proteins.
