# Supplementary material for: A New Master Donor Virus for the Development of Live-Attenuated Influenza B Virus Vaccines
Source: Viruses. 2021 Jun 30;13(7):1278. doi: 10.3390/v13071278 (PMC8310163; doi:10.3390/v13071278)
Supplement: Supplementary file 1 [file viruses-13-01278-s001.zip › viruses-1259865-supplementary.pdf]

Amino acid alignment of B/Ann Arbor/1/1966 and B/Brisbane/60/2008 PB2

```
B/Ann Arbor/1/1966        MTLAKIELLKQLLRDNEAKTVLKQTTVDQYNIIRKFNTSRIERNPSLRMKWAMCSNFPLA
B/Brisbane/60/2008        MTLAKIELLKQLLRDNEAKTVLKQTTVDQYNIIRKFNTSRIEKNPSLRMKWAMCSNFPLA

B/Ann Arbor/1/1966        LTKGDMANRIPLEYKGIRLKTNAEDIGTKGQMCSIAAVTWWNTYGPIGDTEGFEKVYESF
B/Brisbane/60/2008        LTKGDMANRIPLEYKGIQLKTNAEDIGTKGQMCSIAAVTWWNTYGPIGDTEGFERVYESF

B/Ann Arbor/1/1966        FLRKMRLDNATWGRITFGPVERVRKRVLLNPLTKEMPPDEASNVIMEILFPKEAGIPRES
B/Brisbane/60/2008        FLRKMRLDNATWGRITFGPVERVRKRVLLNPLTKEMPPDEASNVIMEILFPKEAGIPRES

B/Ann Arbor/1/1966        TWMHRELIKEKREKLKGTMITPIVLAYMLERELVARRRFLPVAGATSAEFIEMLHCLQGE
B/Brisbane/60/2008        TWIHRELIKEKREKLKGTMITPIVLAYMLERELVARRRFLPVAGATSAEFIEMLHCLQGE

B/Ann Arbor/1/1966        NWRQIYHPGGNKLTESRSQSMIVACRKIVRRSIVASNPLELAVEIANKTVIDTEPLKSCL
B/Brisbane/60/2008        NWRQIYHPGGNKLTESRSQSMIVACRKIIRRSIVASNPLELAVEIANKTVIDTEPLKSCL

B/Ann Arbor/1/1966        AAIDGGDVACDIIRAALGLKIRQRQRFGRLELKRISGRGFKNDEEILIGNGTIQKIGIWD
B/Brisbane/60/2008        AAXDGGDVACDIIRAALGLKIRQRQRFGRLELKRISGRGFKNDEEILIGNGTIQKIGIWD

B/Ann Arbor/1/1966        GEEEFHVRCGECRGILKKSKMRMEKLLINSAKKEDMKDLIILCMVFSQDTRMFQGVRGEI
B/Brisbane/60/2008        GEEEFHVRCGECRGILKKSKMKLEKLLINSAKKEDMRDLIILCMVFSQDTRMFQGVRGEI

B/Ann Arbor/1/1966        NFLNRAGQLLSPMYQLQRYFLNRSNDLFDQWGYEEPPKASELHGINELMNASDYTLKGVV
B/Brisbane/60/2008        NFLNRAGQLLSPMYQLQRYFLNRSNDLFDQWGYEESPKASELHGINESMNASDYTLKGIV

B/Ann Arbor/1/1966        VTKNVIDDFSSTETEKVSITKNLSLIKRTGEVIMGANDVSELESQAQLMITYDTPKMWEM
B/Brisbane/60/2008        VTRNVIDDFSSIETEKVSITKNLSLIKRTGEVIMGANDVSELESQAQLMITYDTPKMWEM

B/Ann Arbor/1/1966        GTTKELVQNTYQWVLKNLVTLKAQFLLGKEDMFQWDAFEAFESIIPQKMAGQYSGFARAV
B/Brisbane/60/2008        GTTKELVQNTYQWVLKNLVTLKAQFLLGKEDMFQWDAFEAFXSIIPQKMAGQYSGFARAV

B/Ann Arbor/1/1966        LKQMRDQEVMKTDQFIKLLPFCFSPPKLRSNGEPYQFLRLMLKGGGENFIEVRKGSPLFS
B/Brisbane/60/2008        LKQMRDQEVMKTDQFIKLLPFCFSPPKLRSNGEPYQFLKLVLKGGGENFIEVRKGSPLFS

B/Ann Arbor/1/1966        YNPQTEVLTICGRMMSLKGKIEDEERNRSMGNAVLAGFLVSGKYDPDLGDFKTIEELEKL
B/Brisbane/60/2008        YNPQTEVLTICGRMMSLKGKIEDEERNRSMGNAVLAGFLVSGKYDPDLGDFKTIEELEKL

B/AnnArbor/1/1966         KPGEKANILLYQGKPVKVVKRKRYSALSNDISQGIKRQRMTVESMGWALS
B/Brisbane/60/2008        KPGEKANILLYQGKPVKVVKRKRYSALSNDISQGIKRQRMTVESMGWALS
```

■ = amino acid identical
□ = amino acid changes
■ (blue) = amino acid changes present in the cold adapted MDV

Amino acid changes:

R43K	R78Q
K115R	M183I
V291T	I303X
R382K	M383L
K397R	P456S
L468S	V479I
K483R	T492I
E582X	R639K
M641V	

Amino acid alignment of B/Ann Arbor/1/1966 and B/Brisbane/60/2008 PB1

B/Ann Arbor/1/1966	MNINPYFLFIDVPIQAAISTTFPYTGVPPYSHGTGTGYTIDTVIRTHEYSNKGKQYISDV
B/Brisbane/60/2008	MNINPYFLFIDVPVQAAISTTFPYTGVPPYSHGTGTGYTIDTVIRTHEYSNKGKQYISDV
B/Ann Arbor/1/1966	TGCAMVDPTNGPLPEDNEPSAYAQLDCVLEALDRMDEEHPGLFQAASQNAMEALMVTTVD
B/Brisbane/60/2008	TGCTMVDPTNGPLPEDNEPSAYAQLDCVLEALDRMDEEHPGLFQAASQNAMEALMVTTVD
B/Ann Arbor/1/1966	KLTQGRQTFDWTVCRNQPAATALNTTITSFRLNDLNGADKGGLVPFCQDIIDSLDKPEMT
B/Brisbane/60/2008	KLTQGRQTFDWTVCRNQPAATALNTTITSFRLNDLNGADKGGLIPFCQDIIDSLDRPEMT
B/Ann Arbor/1/1966	FFSVKNIKKKLPAKNRKGFLIKRIPMKVKDRITRVEYIKRALSLNTMTKDAERGKLKRRA
B/Brisbane/60/2008	FFSVKNIKKKLPAKNRKGFLIKRIPMKVKDKITKVEYIKRALSLNTMTKDAERGKLKRRA
B/Ann Arbor/1/1966	IATAGIQIRGFVLVVENLAKNICENLEQSGLPVGGNEKKAKLSNAVAKMLSNCPPGGISM
B/Brisbane/60/2008	IATAGIQIRGFVLVVENLAKNICENLEQSGLPVGGNEKKAKLSNAVAKMLSNCPPGGISM
B/Ann Arbor/1/1966	TVTGDNTKWNECLNPRIFLAMTERITRDSPIWFRDFCSIAPVLFSNKIARLGKGFMITSK
B/Brisbane/60/2008	TVTGDNTKWNECLNPRIFLAMTERITRDSPVWFRDFCSIAPVLFSNKIARLGKGFMITSK
B/Ann Arbor/1/1966	TKRLKAQIPCPDLFNIPLERYNEETRAKLKKLKPFFNEEGTASLSPGMMMGMFNMLSTVL
B/Brisbane/60/2008	TKRLKAQIPCPDLFSIPLERYNEETRAKLKKLKPFFNEEGTASLSPGMMMGMFNMLSTVL
B/Ann Arbor/1/1966	GVAALGIKNIGNREYLWDGLQSSDDFALFVNAKDEETCMEGINDFYRTCKLLGINMSKKK
B/Brisbane/60/2008	GVAALGIKNIGNKEYLWDGLQSSDDFALFVNAKDEETCMEGINDFYRTCKLLGVNMSKKK
B/Ann Arbor/1/1966	SYCNETGMFEFTSMFYRDGFVSNFAMELPSFGVAGVNESADMAIGMTIIKNNMINNGMGP
B/Brisbane/60/2008	SYCNETGMFEFTSMFYRDGFVSNFAMELPSFGVAGVNESADMAIGMTIIKNNMINNGMGP
B/Ann Arbor/1/1966	ATAQTAIQLFIADYRYTYKCHRGDSKVEGKRMKIIKELWENTKGRDGLLVADGGPNIYNL
B/Brisbane/60/2008	ATAQTAIQLFIADYRYTYKCHRGDSKVEGKRMKIIKELWENTKGRDGLLVADGGPNIYNL
B/Ann Arbor/1/1966	RNLHIPEIVLKYNLMDPEYKGRLLHPQNPFVGHLSIEGIKEADITPAHGPIKKMDYDAVS
B/Brisbane/60/2008	RNLHIPEIVLKYNLMDPEYKGRLLHPQNPFVGHLSIEGIKEADITPAHGPVKKMDYDAVS
B/Ann Arbor/1/1966	GTHSWRTKRNRSILNTDQRNMILEEQCYAKCCNLFEACFNSASYRKPVGQHSMLEAMAHR
B/Brisbane/60/2008	GTHSWRTKRNRSILNTDQRNMILEEQCYAKCCNLFEACFNSASYRKPVGQHSMLEAMAHR
B/Ann Arbor/1/1966	LRMDARLDYESGRMSKDDFEKAMAHLGEIGHI
B/Brisbane/60/2008	LRMDARLDYESGRMSKDDFEKAMAHLGEIGYI

■ = amino acid identical
□ = amino acid changes

Amino acid changes:

I14V	A64T
V164T	K176R
R211K	R214K
I331V	N375S
R433K	I474V
I651V	H751Y

Amino acid alignment of B/Ann Arbor/1/1966 and B/Brisbane/60/2008 PA

```
B/Ann Arbor/1/1966      MDTFITRNFQTTIIQKAKNTMAEFSEDPELQPAMLFNICVHLEVCYVISDMNFLDEEGKT
B/Brisbane/60/2008      MDTFITRNFQTTIIQKAKNTMAEFSEDPELQPAMLFNICVHLEVCYVISDMNFLDEEGKA

B/Ann Arbor/1/1966      YTALEGQGKEQNLRPQYEVIEGMPRNIAWMVQRSLAQEHGIETPRYLADLFDYKTKRFIE
B/Brisbane/60/2008      YTALEGQGKEQNLRPQYEVIEGMPRTIAWMVQRSLAQEHGIETPKYLADLFDYKTKRFIE

B/Ann Arbor/1/1966      VGITKGLADDYFWKKKEKLGNSMELMIFSYNQDYSLSNEHSLDEEGKGRVLSRLTELQAE
B/Brisbane/60/2008      VGITKGLADDYFWKKKEKLGNSMELMIFSYNQDYSLSNESSLDEEGKGRVLSRLTELQAE

B/Ann Arbor/1/1966      LSLKNLWQVLIGEEDIEKGIDFKLGQTISKLRDISVPAGFSNFEGMRSYIDNIDPKGAIE
B/Brisbane/60/2008      LSLKNLWQVLIGEEDVEKGIDFKLGQTISRLRDISVPAGFSNFEGMRSYIDNIDPKGAIE

B/Ann Arbor/1/1966      RNLARMSPLVSVTPKKLKWEDLRPIGPHIYSHELPEVPYNAFLLMSDELGLANMTEGKSK
B/Brisbane/60/2008      RNLARMSPLVSVTPKKLTWEDLRPIGPHIYDHELPEVPYNAFLLMSDELGLANMTEGKSK

B/Ann Arbor/1/1966      KPKTLAKECLEKYSTLRDQTDPILIMKSEKANENFLWKLWRDCVNTISNEETSNELQKTN
B/Brisbane/60/2008      KPKTLAKECLEKYSTLRDQTDPILIMKSEKANENFLWKLWRDCVNTISNEETSNELQKTN

B/Ann Arbor/1/1966      YAKWATGDGLTYQKIMKEVAIDDETMYQEEPKIPNKCRVAAWVQTEMNLLSTLTSKRALD
B/Brisbane/60/2008      YAKWATGDGLTYQKIMKEVAIDDETMCQEEPKIPNKCRVAAWVQTEMNLLSTLTSKRALD

B/Ann Arbor/1/1966      LPEIGPDVAPVEHVGSERRKYFVNEINYCKASTVMMKYVLFHTSLLNESNASMGKYKVIP
B/Brisbane/60/2008      LPEIGPDIAPVEHVGSERRKYFVNEINYCKASTVMMKYVLFHTSLLNESNASMGKYKVIP

B/Ann Arbor/1/1966      ITNRVVNEKGESFDILYGLAVKGQSHLRGDTDVVTVVTFEFSSTDPRVDSGKWPKYTVFR
B/Brisbane/60/2008      ITNRVVNEKGESFDMLYGLAVKGQSHLRGDTDVVTVVTFEFSSTDPRVDSGKWPKYTVFR

B/Ann Arbor/1/1966      IGSLFVSGREKSVYLYCRVNGTNKIQMKWGMEARRCLLQSMQQMEAIVDQESSIQGYDMT
B/Brisbane/60/2008      IGSLFVSGREKSVYLYCRVNGTNKIQMKWGMEARRCLLQSMQQMEAIVEQESSIQGYDMT

B/Ann Arbor/1/1966      KACFKGDRVNSPKTFSIGTQEGKLVKGSFGKALRVIFTKCLMHYVFGNAQLEGFSAESRR
B/Brisbane/60/2008      KACFKGDRVNSPKTFSIGTQEGKLVKGSFGKALRVIFTKCLMHYVFGNAQLEGFSAESRR

B/Ann Arbor/1/1966      LLLLIQALKDRKGPWVFDLEGMYSGIEECISNNPWVIQSAYWFNEWLGFEKEGSKVLESI
B/Brisbane/60/2008      LLLLIQALKDRKGPWVFDLEGMYSGIEECISNNPWVIQSVYWFNEWLGFEKEGNKVLESV

B/Ann Arbor/1/1966      DEIMDE
B/Brisbane/60/2008      DEIMDE
```

= amino acid identical
= amino acid changes
= amino acid changes present in the cold adapted MDV

Amino acid changes:

T60A	N86T
R105K	H160S
I196V	K210R
K258T	S271D
Y387C	V428I
D589E	A700V
S714N	I720V

Amino acid alignment of B/Ann Arbor/1/1966 and B/Brisbane/60/2008 NP

B/Ann Arbor/1/1966	MSNMDIDGINTGTIDKTPEEITSGTSGATRPIIKPATLAPPSNKRTRNPSPERATTSSEA
B/Brisbane/60/2008	MSNMDIDGINTGTIDKTPEEITSGTSGTTRPIIRPATLAPPSNKRTRNPSPERATTSSED
B/Ann Arbor/1/1966	IVGRRTQKKQTPTEIKKSVYNMVVKLGEFYNQMMVKAGLNDDMERNLIQNAHAVERILLA
B/Brisbane/60/2008	DVGRKTQKKQTPTEIKKSVYNMVVKLGEFYNQMMVKAGLNDDMERNLIQNAHAAERILLA
B/Ann Arbor/1/1966	ATDDKKTEYQKKKNARDVKEGKEEIDHNKTGGTFYKMVRDDKTIYFSPIRITFLKEEVKT
B/Brisbane/60/2008	ATDDKKTEFQKKKNARDVKEGKEEIDHNKTGGTFYKMVRDDKTIYFSPIRITFLKEEVKT
B/Ann Arbor/1/1966	MYKTTMGSDGFSGLNHIMIGHSQMNDVCFQRSKALKRVGLDPSLISTFAGSTLPRRSGAT
B/Brisbane/60/2008	MYKTTMGSDGFSGLNHIMIGHSQMNDVCFQRSKALKRVGLDPSLISTFAGSTVPRRSGAT
B/Ann Arbor/1/1966	GVAIKGGGTLVAEAIRFIGRAMADRGLLRDIRAKTAYEKILLNLKNKCSAPQQKALVDQV
B/Brisbane/60/2008	GVAIKGGGTLVAEAIRFIGRAMADRGLLRDIKAKTAYEKILLNLKNKCSAPQQKALVDQV
B/Ann Arbor/1/1966	IGSRNPGIADIEDLTLLARSMVVVRPSVASKVVLPISINAKIPQLGFNVEEYSMVGYEAM
B/Brisbane/60/2008	IGSRNPGIADIEDLTLLARSMVVVRPSVASKVVLPISIYAKIPQLGFNVEEYSMVGYEAM
B/Ann Arbor/1/1966	ALYNMATPVSILRMGDDAKDKSQLFFMSCFGAAYEDQRVLSALTGTEFKPRSALKCKGFH
B/Brisbane/60/2008	ALYNMATPVSILRMGDDAKDKSQLFFMSCFGAAYEDLRVLSALTGTEFKPRSALKCKGFH
B/Ann Arbor/1/1966	VPAKEQVEGMGAALMSIKLQFWAPMTRSGGNEVGGDGGSGQISCSPVFAVERPIALSKQA
B/Brisbane/60/2008	VPAKEQVEGMGAALMSIKLQFWAPMTRSGGNEVGGDGGSGQISCSPVFAVERPIALSKQA
B/Ann Arbor/1/1966	VRRMLSMNIEGRDADVKGNLLKMMNDSMAKKTNGNAFIGKKMFQISDKNKINPVDIPIKQ
B/Brisbane/60/2008	VRRMLSMNIEGRDADVKGNLLKMMNDSMAKKTSGNAFIGKKMFQISDKNKTNPIEIPIKQ
B/Ann Arbor/1/1966	TIPNFFFGRDTAEDYDDLDY
B/Brisbane/60/2008	TIPNFFFGRDTAEDYDDLDY

■ = amino acid identical
□ = amino acid changes
■ (blue) = amino acid changes present in the cold adapted MDV

Amino acid changes:

A28T	K34R
A60D	I61D
R65K	V114A
Y129E	L233V
R272K	N339Y
Q397L	N513S
I531T	V534I
D535E	

Amino acid alignment of B/Ann Arbor/1/1966 and B/Brisbane/60/2008 M1

```
B/Ann Arbor/1/1966      MSLFGDTIAYLLSLTEDGEGKAELAEKLHCWFGGKEFDLDSALEWIKNKRCLTDIQKALI
B/Brisbane/60/2008      MSLFGDTIAYLLSLTEDGEGKAELAEKLHCWFGGKEFDLDSALEWIKNKRCLTDIQKALI

B/Ann Arbor/1/1966      GASICFLKPKDQERKRRFITEPLSGMGTTATKKKGLILAERKMRRCVSFHEAFEIAEGHE
B/Brisbane/60/2008      GASICFLKPKDQERKRRFITEPLSGMGTTATKKKGLILAERKMRRCVSFHEAFEIAEGHE

B/Ann Arbor/1/1966      SSALLYCLMVMYLNPGNYSMQVKLGTLCALCEKQASHSHRAHSRAARSSVPGVRREMQMV
B/Brisbane/60/2008      SSALLYCLMVMYLNPGNYSMQVKLGTLCALCEKQASHSHRAHSRAARSSVPGVRREMQMV

B/Ann Arbor/1/1966      SAMNTAKTMNGMGKGEDVQKLAEELQSNIGVLRSLGASQKNGEGIAKDVMEVLKQSSMGN
B/Brisbane/60/2008      SAMNTAKTMNGMGKGEDVQKLAEELQSNIGVLRSLGASQKNGEGIAKDVMEVLKQSSMGN

B/Ann Arbor/1/1966      SALVKKYL
B/Brisbane/60/2008      SALVKKYL
```

= amino acid identical
= amino acid changes present in the cold adapted MDV

Amino acid changes:
None

Amino acid alignment of B/Ann Arbor/1/1966 and B/Brisbane/60/2008 M2

```
B/Ann Arbor/1/1966      MLEPFQILSICSFILSALHFMAWTIGHLNQIKRGVNLKIRIRNPNKETINREVSILRHSY
B/Brisbane/60/2008      MLEPFQILTICSFILSALHFMAWTIGHLNQIKRGINMKIRIKGPNKETINREVSILRHSY

B/Ann Arbor/1/1966      QKEIQAKETMKEVLSDNMEILSDHIVIEGLSAEEIIKMGETVLEVEELQ
B/Brisbane/60/2008      QKEIQAKETMKEVLSDNMEVLNDHIIIEGLSAEEIIKMGETVLEIEELH
```

■ = amino acid identical

□ = amino acid changes

Amino acid changes:

S9T	V35I
L37M	R42K
N43G	I80V
S82N	V86I
V105I	Q109H

Amino acid alignment of B/Ann Arbor/1/1966 and B/Brisbane/60/2008 NS1

```
B/Ann Arbor/1/1966      MAD-NMTTTQIEVGPGATNATINFEAGILECYERLSSQRALDYPGQDRLNRLKRKLESRI
B/Brisbane/60/2008      MANNNMTTTQIEVGPGATNATINFEAGILECYERLSWQRALDYPGQDRLNRLKRKLESRI

B/Ann Arbor/1/1966      KTHNKSEPESKRMSLEERKAIGVKMMKVLLFMNPSAGIEGFEPYCMKNSSNSNCPNCNWT
B/Brisbane/60/2008      KTHNKSEPESKRMSLEERKAIGVKMMKVLLFMNPSAGIEGFEPYCMKSSSNSNCTKYNWT

B/Ann Arbor/1/1966      DYPPTPGKCLDDIEEEPENVDDPTEIVLRDMNNKDARQKIKEEVNTQKEGKFRLTIKRDI
B/Brisbane/60/2008      DYPSTPERCLDDIEEEPEDVDGPTEIVLRDMNNKDARQKIKEEVNTQKEGKFRLTIKRDM

B/Ann Arbor/1/1966      RNVLSLRVLVNGTFLKHPNGYKSLSTLHRLNAYDQSGRLVAKLVATDDLTVEDEEDGHRI
B/Brisbane/60/2008      RNVLSLRVLVNGTFLKHPNGHKSLSTLHRLNAYDQSGRLVAKLVATDDLTVEDEEDGHRI

B/Ann Arbor/1/1966      LNSLFERFNEGHSKPIRAAETAVGVLSQFGQEHRLSPEEGDN
B/Brisbane/60/2008      LNSLFERLNEGHSKPIRAAETAVGVLSQFGQEHRLSPEEGDN
```

■ = amino acid identical

□ = amino acid changes

Amino acid changes:

D3N	Δ4N
S37W	N107S
P114T	N115K
C116Y	P123S
G126E	K127R
N138D	D141G
Y200H	F247L

Amino acid alignment of B/Ann Arbor/1/1966 and B/Brisbane/60/2008 NEP

```
B/Ann Arbor/1/1966      MAD-NMTTTQIEWRMKKMAIGSSTHSSSVLMKDIQSQFEQLKLRWESYPNLVKSTDYHQR
B/Brisbane/60/2008      MANNNMTTTQIEWRMKKMAIGSSTHSSSVLMKDIQSQFEQLKLRWESYPNLVKSTDYHQK

B/Ann Arbor/1/1966      RETIRLVTEELYLLSKRIDDNILFHKTVIANSSIIADMIVSLSLLETLYEMKDVVEVYSR
B/Brisbane/60/2008      RETIRLVTEELYLLSKRIDDNILFHKTVIANSSIIADMVVSLSLLETLYEMKDVVEVYSR

B/Ann Arbor/1/1966      QCL
B/Brisbane/60/2008      QCL
```

■ = amino acid identical
□ = amino acid changes

Amino acid changes:

D3N	Δ4N
R60K	I99V
